# Endothelial Contributions to Zebrafish Heart Regeneration

**DOI:** 10.3390/jcdd5040056

**Published:** 2018-12-11

**Authors:** Cristina E. Fernandez, Melanie Bakovic, Ravi Karra

**Affiliations:** 1 Department of Medicine, Duke University Medical Center, Durham, NC 27710, USA; cef19@duke.edu (C.E.F.); melanie.bakovic@duke.edu (M.B.); 2 Department of Biomedical Engineering, Duke University Medical Center, Durham, NC 27708, USA; 3 Regeneration Next, Duke University, Durham, NC 27710, USA

**Keywords:** cardiac regeneration, vascular, endothelium

## Abstract

Studies over the past two decades have shown heart regeneration in zebrafish to be a dynamic process, choreographed by multiple cell types. In particular, recent work has identified revascularization of the wound to be a sentinel event during heart regeneration. The cardiac endothelium has emerged as a key orchestrator of heart regeneration, influencing cardiomyocyte hyperplasia and tissue morphogenesis. Here, we review how the coronary vasculature regenerates after injury, how signaling pathways link the cardiac endothelium to heart regeneration, and how understanding these signaling dynamics can lead to targeted therapies for heart regeneration.

Approximately 250,000–500,000 Americans suffer from advanced heart failure refractory to guideline-directed medical therapy [1]. These patients have a one-year mortality approaching 80%, rivaling the most virulent cancers [2]. By replacing lost or dysfunctional cardiac tissue, therapeutic heart regeneration has the potential for reversing heart failure and could be transformational. Over the past two decades, seminal work on the mechanisms of innate heart regeneration in model systems (such as zebrafish) has moved the concept of therapeutic heart regeneration away from science fiction to the verge of clinical investigation. Lessons learned about regenerative biology will need to be carefully considered when attempting translational studies.

Tissue revascularization is of particular relevance toward achieving therapeutic heart regeneration. A large fraction of patients with advanced heart failure suffer from ischemic heart disease, meaning that the injured myocardium is poorly perfused. Thus, how the vasculature influences the regenerative landscape of the heart is of critical importance. Here, we provide a focused overview of how the coronary vasculature regenerates after injury and the signaling pathways that link the cardiac endothelium to heart regeneration. For the purpose of this review, we consider the endocardium and the coronary vascular endothelium to make up the cardiac endothelium, as these endothelial populations have a similar ontogeny and highly overlapping marker profiles.

## 1. Cellular Mechanisms of Endogenous Heart Regeneration

**Figure 1:**
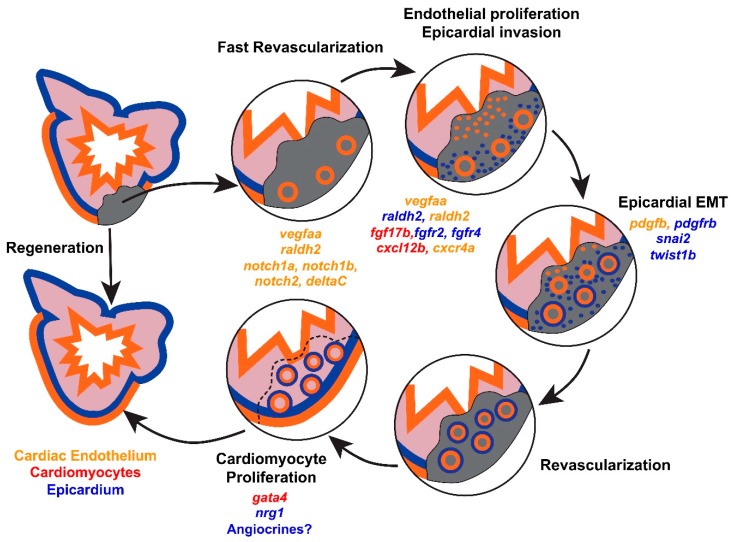
**Cellular dynamics during zebrafish heart regeneration.** Immediately after injury, fast revascularization of the wound occurs by sprouting of rudimentary vasculature into the wound. Shortly thereafter, the cardiac endothelium (orange) and epicardium (blue) proliferate and invade the injured area. Epicardial cells undergo EMT and support revascularization. Subsequently, cardiomyocyte (red) proliferation occurs leading to newly formed myocardium. Selected modifiers of regenerative responses are provided for each stage of regeneration, color coded by the likely cellular source.

Following cardiac injury, axolotls and zebrafish are able to replace lost cardiac tissue through tissue regeneration [3,4,5]. Lineage tracing studies have revealed the cellular source of new cardiomyocytes to be primarily pre-existing cardiomyocytes, as opposed to the expansion and differentiation of progenitor cells [6,7]. Mechanistically, spared cardiomyocytes lose sarcomeric structures, upregulate developmental transcription factors such as *gata4*, and re-enter the cell cycle (Figure 1) [6,7,8]. In regenerative organisms, the capacity for cardiomyocyte proliferation is robust. Following genetic ablation of over 60% of cardiomyocytes in zebrafish, up to 44% of the remaining cardiomyocytes re-enter the cell cycle [9]. By contrast, non-regenerative organisms have a limited capacity for cardiomyocyte hyperplasia after injury [10]. Based on these observations, stimulating cardiomyocyte proliferation has emerged as a focal point for methods to enhance the regenerative capacity of the mammalian heart [11,12]. However, innate heart regeneration is a complex process, dependent on contributions from multiple cell types.

Among the earliest responses to cardiac injury in zebrafish is activation of the cardiac endothelium and the overlying epicardium [8,13]. Within hours of cryoinjury, rudimentary vessels invade the wound (Figure 1). Whole-mount imaging of *fli1a*:*EGFP* fish, at fine intervals following cryoinjury, suggests that these vessels sprout from the coronary vasculature [14]. Later in regeneration, the overlying epicardium proliferates and infiltrates the wound (Figure 1) [14]. Epicardial responses are critical to zebrafish heart regeneration, and have been reviewed in detail [15,16]. Briefly, epicardial cells undergo epithelial to mesenchymal transition (EMT) to generate mural cells and fibroblasts, and support a regenerative milieu by influencing the production of hydrogen peroxide and paracrine growth factors [17,18,19,20]. Interference with revascularization responses, epicardial responses, or fibroblast responses limits the extent of cardiomyocyte proliferation and heart regeneration [18,20].

In addition to resident cardiac tissues, inflammatory cells enable heart regeneration. Limiting innervation of the wound by overexpression of the neuro-repellant *sema3aa* severely delays regeneration, possibly by altering the inflammatory response [21]. In turn, the quality of the inflammatory response is likely to influence regeneration by facilitating a regenerative milieu. Specialized regulatory T-cells that infiltrate the wound express tissue-specific mitogens in response to injury [22]. Similarly, macrophages are likely to have pro-regenerative paracrine effects [23]. Together, this wealth of data highlights the concerted and complex contributions of multiple tissues for natural heart regeneration.

## 2. Coronary Revascularization during Heart Regeneration

Mechanistic studies over the past several years have demonstrated that hindrance of revascularization disrupts heart regeneration. In most cases, interference with revascularization is also associated with defects in cardiomyocyte proliferation. Here, we summarize key elements for myocardial revascularization and regeneration.

### 2.1. Dynamic Remodeling of the Cardiac Endothelium after Injury

Regenerative organisms respond to cardiac injury with robust revascularization. By contrast, ischemic injuries in mammals result in persistent tissue perfusion defects. Thus, an important feature of regeneration is revascularization of the wound. Cardiac endothelial responses and angiogenesis during heart regeneration have been best characterized in zebrafish [14,24]. Within hours of cryoinjury, the cardiac endothelium undergoes a transition from thin, elongated cells that are tightly coupled to neighboring cardiomyocytes, to a more rounded and detached morphology [24]. These morphologic changes initially occur throughout the heart, but localize to the site of injury during regeneration [13,24]. Such morphological changes may permit endothelial expansion and even migration, as filipodia are present in the regenerating endocardium [24]. Coincident with a changing morphology, endocardial cells upregulate developmental factors, such as the transcription factors Erg and *nfatc1a* [24].

Formal lineage tracing studies in regenerating zebrafish hearts have confirmed that revascularization occurs primarily by angiogenesis (Figure 1). Using a *kdrl*:*CreER*; *ubi*:*Switch* bi-transgenic fish, Zhao, et al. were able to genetically label the cardiac endothelium and confirm that the new vasculature during regeneration is derived from pre-existing cardiac endothelium [25]. Because Zhao, et al. induced recombination in embryos to achieve widespread labeling of the adult cardiac endothelium, the precise endothelial population that contributes to coronary revascularization has yet to be determined. Interestingly, following cryoinjury, nascent vascular sprouts do not initially express markers of mature arterial (*kdrl* and *dll4in3*), venous (*flt4*), or lymphatic (*flt4*) endothelium [14]. However, cryoinjury of the endocardial enhancer trap line *ET33mi60* suggests that rudimentary vessels express endocardial markers, although a detailed analysis of the time points corresponding to the earliest vascular sprouts was not reported [24]. Consistent with endocardial expansion, the endocardium expresses markers of cell-cycle reentry, with a peak in proliferation that occurs prior to the peak in cardiomyocyte proliferation [24]. Thus, nascent revascularization of the wound may provide instructive cues for heart regeneration, or potentially act as a scaffold for regenerative growth (Figure 1).

### 2.2. vegfaa Signaling

Two recent studies have described the role of *vegfaa* in promoting angiogenesis during heart regeneration (Table 1) [14,26]. After cryoinjury, *vegfaa* is sharply upregulated, with an expression peak at 1 day post-injury, as measured by quantitative PCR [14]. Using a transgenic reporter strain, we found *vegfaa* expression in the epicardium under homeostatic conditions and additional expression in endocardial cells adjacent to the wound after resection of the ventricular apex (Figure 1) [26]. This expression domain of *vegfaa* in zebrafish appears to differ from mammals, as the *vegfaa* ortholog *Vegfa* is induced in cardiomyocytes after injury. Although zebrafish *vegfaa* mutants regenerate normally, inhibition of *vegfaa* signaling, by expression of a dominant negative version of *vegfaa*, blocks regeneration (Table 1) [14]. This block is associated with a 75% reduction in the vascular response to injury, and a 60% reduction in the fraction of proliferating cardiomyocytes [14]. Importantly, solely inhibiting the early revascularization response, without affecting later revascularization, is enough to abrogate zebrafish heart regeneration [14]. Together, these studies indicate that injury-induced *vegfaa* is required for both revascularization and cardiomyocyte proliferation. Of note, the receptors for *vegfaa*, *flt1* and *flk1*, are exclusively expressed by the cardiac endothelium, suggesting that *vegfaa* effects on cardiomyocyte proliferation are mediated by the cardiac endothelium [14,27].

### 2.3. cxcl12b/cxcr4a Signaling

In zebrafish, the coronary vasculature develops as the compact muscle expands during juvenile growth, offering an opportunity to dissect mechanisms of coronary vessel development over several weeks. Harrison, et al. were able to take advantage of this developmental window to identify a *cxcl12b*/*cxcr4a* axis that regulates coronary sprouting from the endocardium. *cxcl12b* is expressed by ventricular cardiomyocytes, in a graded fashion that correlates with the emergence of the coronary vasculature [27]. The endothelium of sprouting vessels, in turn, expresses *cxcr4a*, the receptor for *cxcl12b*. During regeneration, the newly formed vasculature reexpresses *cxcr4a*, suggesting that developmental mechanisms for coronary angiogenesis are reactivated during regeneration [14]. Importantly, mutant *cxcr4a*^−/−^ fish have a markedly diminished coronary vasculature, permitting assay of regenerative responses in the absence of the coronary system [27]. After apical resection, *cxcr4a*^−/−^ fish have gross defects in cardiac regeneration with increased scarring at the site of injury, demonstrating that the coronary vasculature is a necessary participant in guiding and coordinating regeneration [27].

### 2.4. Epicardial Influences on Angiogenesis

The epicardium is the outer mesothelial covering of the heart. During heart development, the epicardium is multipotent, with contributions to perivascular cells, fibroblasts, and, to a lesser extent, other cell types [28,29,30]. Additionally, the epicardium serves a paracrine role to regulate cardiomyocyte proliferation and development of the coronary vasculature [31,32]. During zebrafish heart regeneration, the epicardium has analogous roles. In particular, the epicardium supports coronary vascular homeostasis and facilitates revascularization (Figure 1).

During zebrafish heart development, fibroblast growth factor (FGF) signaling establishes the number of cardiomyocytes, and influences heart size and chamber identity [33,34]. FGF signaling is also critical to heart regeneration (Table 1) [8]. An in situ hybridization survey of FGF ligands during zebrafish heart regeneration revealed *fgf17b* to be upregulated following injury, particularly in the myocardium adjacent to the wound. In parallel, the FGF receptors, *fgfr2* and *fgfr4*, are expressed by a subset of epicardial-derived cells at the wound. Perturbation of FGF signaling during heart regeneration, by expression of a *dn-fgfr1* throughout the heart, abrogates regeneration; demonstrating an inherent requirement of FGF signaling for heart regeneration. Mechanistically, inhibition of FGF signaling is associated with impaired infiltration of *tbx18*^+^ epicardial cells into the wound and reduced coronary revascularization [8]. As FGF receptors are primarily expressed in epicardial cells, this study was among the first to suggest that regenerative revascularization depends on signals from the overlying epicardium.

In addition to invasion of the wound, epicardial cells undergo epithelial to mesenchymal transition (EMT) and contribute to an extensive network of perivascular cells during heart regeneration (Figure 1) [35]. *pdgfrb* has been used to identify these new perivascular cells, and implicates a dynamic platelet derived growth factor (PDGF) signaling axis during heart regeneration that mediates epicardial EMT. Supporting this notion, treatment of zebrafish cardiac explants with PDGF-B induces loss of an epithelial phenotype, including induction of stress fibers and loss of ZO-1 tight junctions. Moreover, pharmacologic inhibition of PDGF signaling after resection of the ventricular apex in zebrafish reduces the expression of EMT markers, and decreases neovascularization of the wound by about 9-fold [35]. Based on this work, perivascular cells are likely to be a critical subset of epicardial-derived cells that support regenerative revascularization.

## 3. Endothelial Influences on Cardiomyocyte Hyperplasia

The spatiotemporal coupling of coronary artery development with cardiomyocyte expansion strongly suggests that the cross-talk between cardiomyocytes and the cardiac endothelium motivates cardiac growth. Recent work indicates that activation of endothelium may be sufficient for heart regeneration, and argues for the presence of angiocrine effectors of heart regeneration.

### 3.1. Retinoic Acid Signaling

In the zebrafish embryo, retinoic acid signaling has critical roles in the specification of cardiac progenitors and patterning of the heart [36,37]. *Raldh2* enzymatically oxidizes retinaldehyde to make retinoic acid, and is a key regulator of tissue levels of retinoic acid. Within 1 h of resection of the adult zebrafish ventricular apex, *raldh2* is induced in the atrial endocardium, with a more global upregulation throughout the endocardium by 6 h. By 24 h, endocardial *raldh2* localizes to the wound (Figure 1) [13]. While the epicardium also upregulates *raldh2*, epicardial cells that upregulate *raldh2* are not abundantly present in the wound until 3–7 days post-amputation, suggesting that early levels of retinoic acid during heart regeneration are regulated by the cardiac endothelium [8,13]. Inhibition of retinoic acid signaling after cardiac injury, by overexpression of a dominant negative version of RAR-alpha or *cyp26a1*, an enzyme that degrades retinoic acid, impairs cardiomyocyte proliferation by more than 6-fold (Table 1). Conversely, treatment of injured zebrafish with a retinoid agonist fails to enhance cardiomyocyte proliferation, suggesting that retinoic acid serves a permissive role during heart regeneration [13]. Intriguingly, *raldh2* is not rapidly upregulated by the cardiac endothelium of mice following injury, raising the possibility that endocardial retinoic acid production plays a role in establishing regenerative capacity [13].

### 3.2. Notch Signaling

Notch signaling is one of the most extensively described pathways activated during heart regeneration (Table 1). Increases in the expression of Notch pathway members after apical resection of the zebrafish heart were first reported following an in situ hybridization screen for factors specific to heart regeneration. Both *notch1b* and the Notch ligand *deltaC* were noted to be upregulated in the endocardium adjacent to the wound [38]. Two more recent in situ hybridization surveys of Notch pathway members have shown *notch1a*, *notch1b*, *notch2*, and *notch3* to be lowly expressed in the endocardium under homeostatic conditions [24,25]. However, only *notch1a*, *notch1b*, and *notch2* are upregulated in the endocardium after amputation (Figure 1). In the epicardium, *notch1a*, *notch2*, and *notch3* are expressed, with *notch1a* and *notch2* expression increasing after injury. While none of these studies detected expression of Notch pathway members in cardiomyocytes, a reporter for Notch activity showed activation in cardiomyocytes during development [39]. Furthermore, functional manipulation of Notch activity during development has revealed an inverse relationship between myocardial Notch activity and trabeculation. Thus, while Notch activity is likely to cell-autonomously modulate endocardial and epicardial responses during heart regeneration, cell-autonomous effects in cardiomyocytes are also possible.

Functional evaluation of Notch signaling after cardiac injury in zebrafish has revealed a vital role during heart regeneration. Inhibition of Notch activity by administration of a γ-secretase inhibitor, or by overexpression of a dominant-negative isoform of the murine mastermind-like protein (*DN-MAML*), results in defective heart regeneration [24,25]. In both instances, attenuated Notch activity is associated with a reduced fraction of proliferating cardiomyocytes, while gross revascularization of the injured area remains intact. To assess the effects of increased Notch activity, transgenic over-expression of the *notch1a*-intracellular domain (*NICD*) has been tested after cryoinjury and apical resection [24,25]. While heart regeneration is limited in both contexts, cardiomyocyte proliferation decreases after resection injury, but increases after cryoinjury. The reasons for these discrepant results are not clear, but could be related to the mode of injury or the heat-shock protocol needed for *NICD* overexpression. Mechanistically, manipulation of Notch activity is suggested to be independent of epicardial responses, as the epicardium is able to infiltrate the wound following *DN-MAML* overexpression [25]. Instead, Notch may exert its effects by modulating endocardial maturation and the inflammatory response. Through gain and loss of function experiments of Notch activity, Munch, et al. associated reduced levels of Notch activity with increased inflammation and reduced maturation of the cardiac endothelium [24]. Additional work with cell-type specific tools for manipulating Notch activity will be needed to dissect cell-autonomous effects of Notch activation during heart regeneration.

### 3.3. vegfaa Signaling

Based on the observation that cardiomyocyte hyperplasia follows expansion of the coronary vasculature during cardiac growth and regeneration, our group generated a transgenic zebrafish line to conditionally overexpress *vegfaa* from cardiomyocytes, in order to evaluate how activation of the vasculature affects cardiac growth (Table 1) [26]. We chose to overexpress *vegfaa* because *vegfaa* is considered to be a master angiogenic factor and its receptors, *flt1* and *kdrl*, are predominantly expressed by endothelial cells in zebrafish. Furthermore (as described above), *vegfaa* is dynamically induced in the endocardium adjacent to the wound following injury, providing physiologic relevance for studies of the *vegfaa* ligand.

At all stages of development in zebrafish, we found that *vegfaa* could stimulate ectopic expansion of the coronary vasculature [26]. Somewhat unexpectedly, however, we also noted that *vegfaa* overexpression also results in cardiomyocyte hyperplasia. In fact, *vegfaa* overexpression is sufficient to induce an entire ectopic regenerative program with activation of the endocardium, epicardium, and myocardium. These phenotypes are reminiscent of those observed with overexpression of the cardiomyocyte mitogen, *nrg1* [40]. Indeed, we noted that *vegfaa* regulatory sequences are activated in the context of *nrg1* overexpression, consistent with recent work in developing mice that *Vegfa* may be downstream of Nrg1 signaling [26,41].

In addition to studying *vegfaa* signaling under homeostatic conditions, we also examined the effects of global *vegfaa* overexpression following resection of the ventricular apex [26]. Consistent with our results in uninjured zebrafish hearts, we noted increases in cardiomyocyte proliferation, both at the wound and away from the wound. However, we unexpectedly noted that regeneration was impaired with *vegfaa* overexpression, specifically with cardiac growth occurring throughout the heart, but not specifically at the injury site. The discrepancy between increased cardiomyocyte proliferation and impaired regeneration are strongly suggestive that *vegfaa* signaling, and the resulting angiogenesis, have a role in directing cardiac growth and morphogenesis during heart regeneration. Additional work to identify the precise angiocrine factors that mediate effects of *vegfaa* is needed.

## 4. Implications for Therapeutic Heart Regeneration

For patients with advanced heart failure, recovery of functional myocardium has long been a major goal. To date, approaches to replenish functional cardiac tissue have included the use of progenitor cells, injection of viruses to restore cardiomyocyte function, injection of biomaterials to reduce ventricular wall stress, and injection of growth factors. Unfortunately, in all instances thus far, progress has been limited.

Seminal work over the past decade has identified a very limited, but present, capacity for cardiomyocyte turnover in the adult human heart, raising the possibility that cardiomyocyte expansion can be leveraged for heart regeneration similar to zebrafish [42,43]. Indeed, many of the mechanisms for innate heart regeneration are conserved from fish to mammals. Neonatal mice are able to regenerate their hearts after injury by cardiomyocyte hyperplasia, coronary revascularization, epicardial expansion, and a reparative inflammatory response [44,45,46]. This capacity for regeneration is lost in adult mammals, in part, because of a diminished ability for cardiomyocyte cell-cycle reentry [45]. However, comparative studies of fish and adult mammals have provided clues for augmenting the regenerative potential of the adult mammalian heart. For example, zebrafish cardiomyocytes are relatively hypoxic, compared to adult mammalian cardiomyocytes [44,47,48]. Accordingly, hypoxic conditioning of the adult mammalian heart improves regenerative capacity [44].

Work in regenerative model systems may also help to refine prior attempts at therapeutic cardiac repair. Of particular relevance to this review is the clinical experience of overexpressing *Vegfa* in the human heart. While pre-clinical studies of the mammalian heart have demonstrated enhanced recovery with exogenous *Vegfa*, human studies have not been as rewarding [49,50]. A major reason for the lack of VEGFA efficacy in early human studies may be under-dosing of VEGFA due to dose-limiting side-effects [49]. Our work in zebrafish suggests that a threshold level of *Vegfa* is needed to stimulate cardiac growth, as *vegfaa* is expressed at low levels in the uninjured heart, but overexpression of *vegfaa* above this level results in ectopic cardiomyogenesis [26]. Additionally, our work in zebrafish suggests that the site of *Vegfa* overexpression is critical to growth effects, as mis-expression during heart regeneration results in unwanted growth away from the site of injury. Finally, more mechanistic work in regenerative organisms is needed to identify the precise angiocrine factors that coordinate heart regeneration. Such work may result in novel factors that can be used to stimulate regenerative growth without dose-limiting side effects.

## Figures and Tables

**Table 1:** Key Pathways Affecting Endothelial Signaling in Zebrafish. FGF, fibroblast growth factor; CM, cardiomyocyte; DN-MAML, dominant-negative isoform of the murine mastermind-like protein; NICD, *notch1a*-intracellular domain; PDGF, platelet-derived growth factor; RA, retinoic acid; EMT, epithelial to mesenchymal transition.

Pathway	Factors Induced after Injury	Functional Evidence during Heart Regeneration	Refs.
FGF	Epicardium: *fgfr2*, *fgfr4* Cardiomyocytes: *fgf17b*	Global expression of *dn-fgfr1* impairs coronary revascularization, epicardial infiltration, and regeneration.	[8]
Notch	Endocardium: *notch1a*, *notch1b*, *deltaC* Epicardium: *notch1a*, *notch2*	Notch inhibition by a γ-secretase inhibitor impairs CM proliferation, endocardial maturation, and regeneration.	[24]
		Notch inhibition by global expression of DN-MAML impairs CM proliferation and regeneration.	[24,25]
		Notch activation by global expression of NICD impairs regeneration. NICD overexpression increases CM proliferation after cryoinjury, but decreases CM proliferation after amputation.	[24,25]
PDGF	Perivascular cells: *pdgfrβ*	Pharmacologic inhibition of PDGF signaling impairs revascularization after amputation.	[35]
RA	Epicardium and endocardium: *raldh2*	RA inhibition by global expression of *dn-rar* or *cyp26a1* impairs CM proliferation. RA activation with a retinoid agonist has no effect on CM proliferation.	[13]
VEGFA	Epicardium and endocardium: *vegfaa*	VEGFA inhibition by global expression of *dn-vegfaa* impairs revascularization, CM proliferation, and regeneration. *vegfaa* overexpression promotes vascular expansion, epicardial EMT, and CM proliferation.	[14] [26]
